# Supplementary material for: Major waves of H2A.Z incorporation during mouse oogenesis and preimplantation embryo development
Source: Nat Commun. 2025 Dec 2;17:210. doi: 10.1038/s41467-025-66919-x (PMC12779981; doi:10.1038/s41467-025-66919-x)
Supplement: Supplementary file 1 — Supplementary Information [file 41467_2025_66919_MOESM1_ESM.pdf]

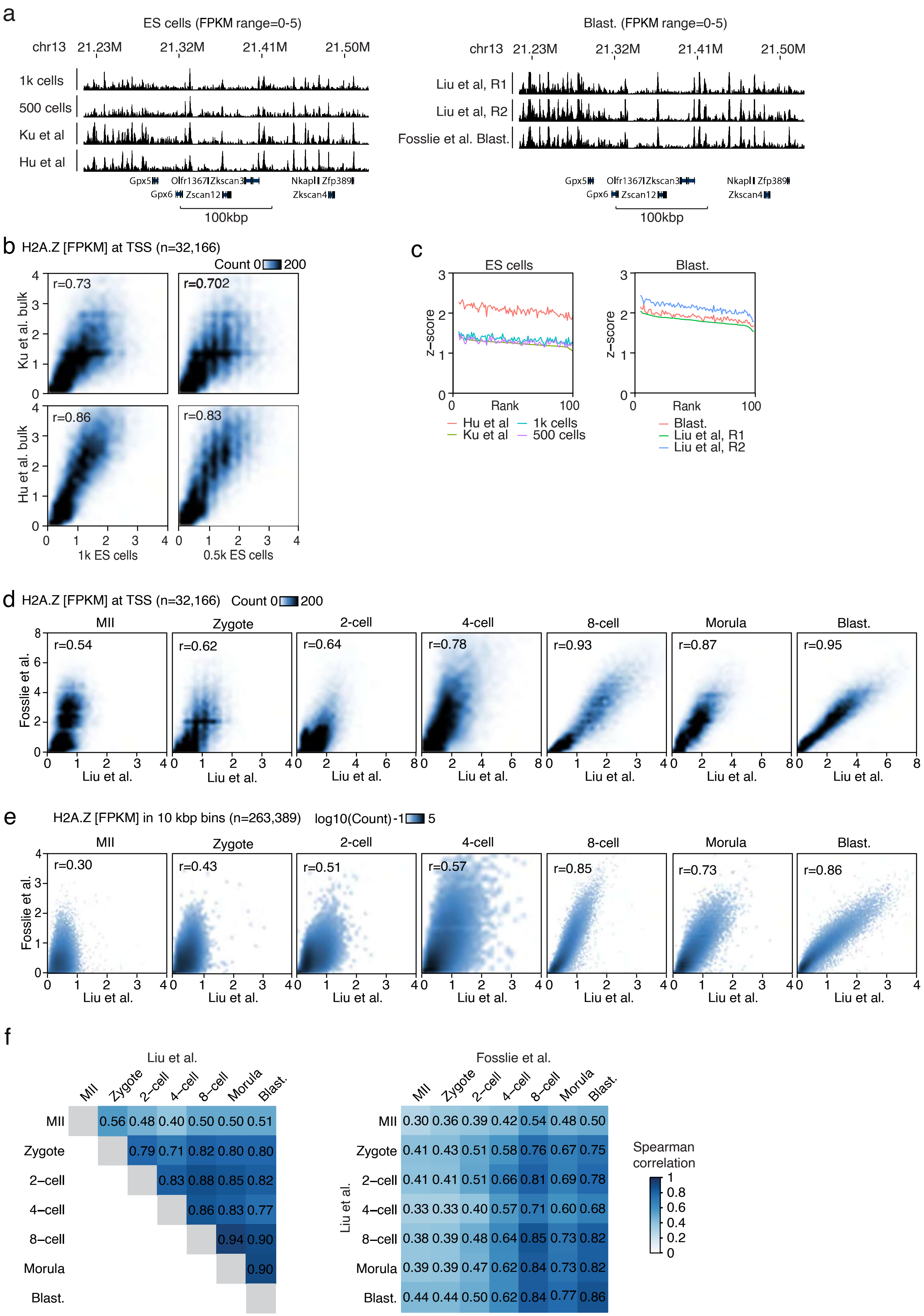
a
ES cells (FPKM range=0-5)
chr13 21.23M 21.32M 21.41M 21.50M
1k cells
500 cells
Ku et al
Hu et al
Gpx5 Olfr1367 Zkscan3 Nkapl Zfp389
Gpx6 Zscan12 Zkscan4
100kbp
Blast. (FPKM range=0-5)
chr13 21.23M 21.32M 21.41M 21.50M
Liu et al, R1
Liu et al, R2
Fosslie et al. Blast.
Gpx5 Olfr1367 Zkscan3 Nkapl Zfp389
Gpx6 Zscan12 Zkscan4
100kbp
b H2A.Z [FPKM] at TSS (n=32,166)
Count 0 200
r=0.73
r=0.702
r=0.86
r=0.83
Ku et al. bulk
Hu et al. bulk
1k ES cells
0.5k ES cells
c
ES cells
Blast.
z−score
Rank
Hu et al
1k cells
Ku et al
500 cells
Blast.
Liu et al, R1
Liu et al, R2
d H2A.Z [FPKM] at TSS (n=32,166) Count 0 200
MII Zygote 2-cell 4-cell 8-cell Morula Blast.
r=0.54 r=0.62 r=0.64 r=0.78 r=0.93 r=0.87 r=0.95
Fosslie et al.
Liu et al.
e H2A.Z [FPKM] in 10 kbp bins (n=263,389) log10(Count)-1 5
MII Zygote 2-cell 4-cell 8-cell Morula Blast.
r=0.30 r=0.43 r=0.51 r=0.57 r=0.85 r=0.73 r=0.86
Fosslie et al.
Liu et al.
f
Liu et al.
MII Zygote 2−cell 4−cell 8−cell Morula Blast.
MII 0.56 0.48 0.40 0.50 0.50 0.51
Zygote 0.79 0.71 0.82 0.80 0.80
2−cell 0.83 0.88 0.85 0.82
4−cell 0.86 0.83 0.77
8−cell 0.94 0.90
Morula 0.90
Blast.
Fosslie et al.
MII Zygote 2−cell 4−cell 8−cell Morula Blast.
Liu et al.
MII 0.30 0.36 0.39 0.42 0.54 0.48 0.50
Zygote 0.41 0.43 0.51 0.58 0.76 0.67 0.75
2−cell 0.41 0.41 0.51 0.66 0.81 0.69 0.78
4−cell 0.33 0.33 0.40 0.57 0.71 0.60 0.68
8−cell 0.38 0.39 0.48 0.64 0.85 0.73 0.82
Morula 0.39 0.39 0.47 0.62 0.84 0.73 0.82
Blast. 0.44 0.44 0.50 0.62 0.84 0.77 0.86
Spearman correlation
1 0.8 0.6 0.4 0.2 0

Supplementary Fig. 1 | High concordance of embryonic H2A.Z profiles with previously published data

a, Genome tracks of H2A.Z ChIP-seq signal from this study compared to previously published H2A.Z ChIP-seq signal from ES cells[14,26] and blastocysts (blast.)[15]. **b**, 2D-histograms showing a comparison between H2A.Z enrichment in ES cells from this study to ES cells from[14,26] at TSSs (n=32,166) ±1kbp. r: Spearman's rank correlation coefficients. **c**, Z-scores of H2A.Z signal at TSSs (±1kbp) in ES cell[14,26] and blastocyst samples[15]. Z-scores are ranked based on[15,26] R1 samples in the respective plots. **d**, **e**, 2D-histograms showing the comparison between H2A.Z enrichment at different developmental stages from our study (Y-axis) and previously published samples (X-axis)[15] at unique TSS (**d**) (n=32,166) ±1kbp, and throughout the genome divided into 10 kbp bins (**e**) (n=263,389). r: Spearman's rank correlation coefficients; Blast., blastocyst. **f,** Correlation matrices of H2A.Z enrichment throughout the whole genome, measured by FPKM values in 10 kb genomic bins. Left: correlations between samples from the same study. Right: correlations between samples from different studies.

Fig. S2

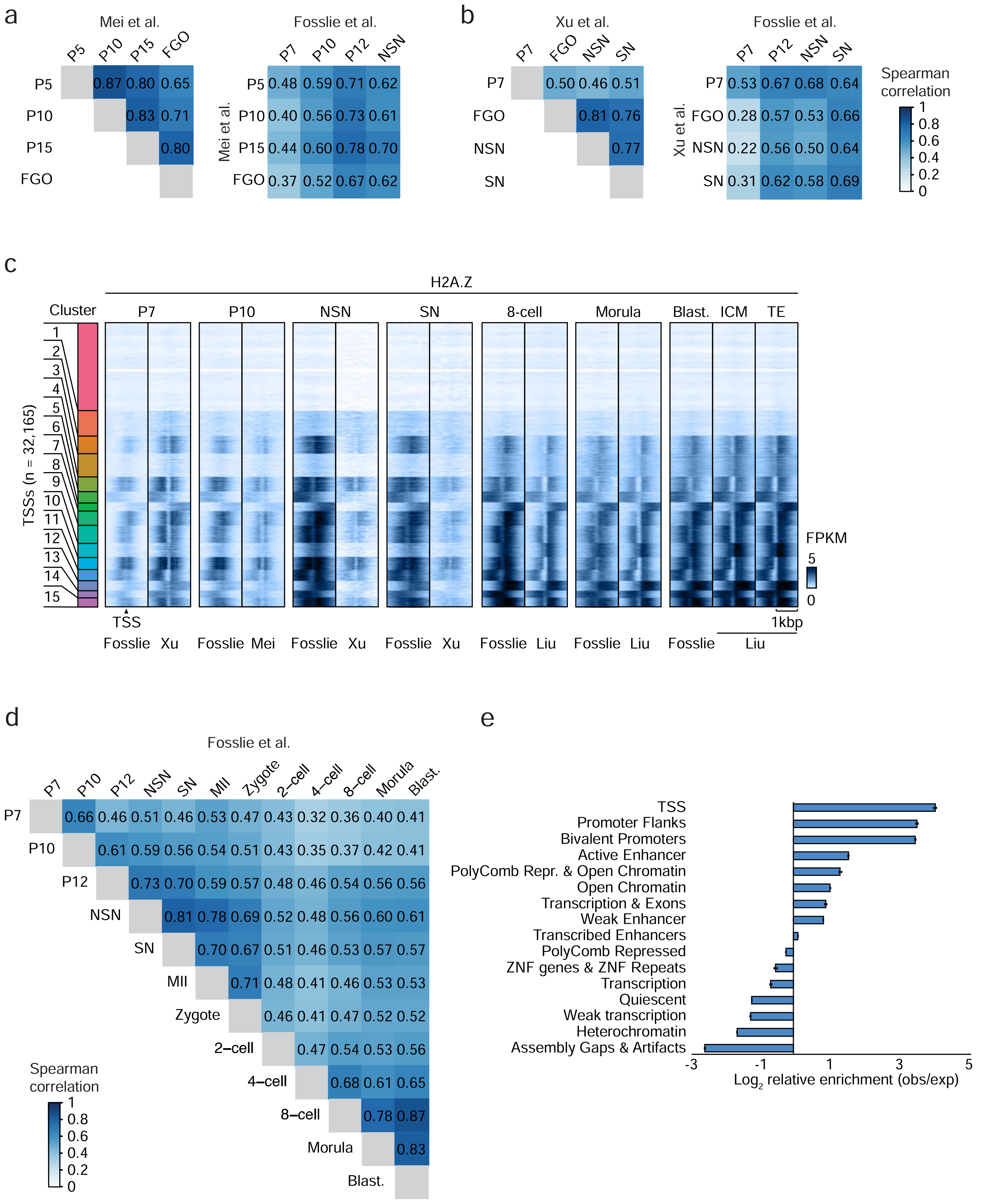

a
Mei et al.
P5 P10 P15 FGO
P5 0.87 0.80 0.65
P10 0.83 0.71
P15 0.80
FGO
Fosslie et al.
P7 P10 P12 NSN
Mei et al.
P5 0.48 0.59 0.71 0.62
P10 0.40 0.56 0.73 0.61
P15 0.44 0.60 0.78 0.70
FGO 0.37 0.52 0.67 0.62
b
Xu et al.
P7 FGO NSN SN
P7 0.50 0.46 0.51
FGO 0.81 0.76
NSN 0.77
SN
Fosslie et al.
P7 P12 NSN SN
Xu et al.
P7 0.53 0.67 0.68 0.64
FGO 0.28 0.57 0.53 0.66
NSN 0.22 0.56 0.50 0.64
SN 0.31 0.62 0.58 0.69
Spearman correlation
1 0.8 0.6 0.4 0.2 0
c
H2A.Z
Cluster
P7 P10 NSN SN 8-cell Morula Blast. ICM TE
1 2 3 4 5 6 7 8 9 10 11 12 13 14 15
TSSs (n = 32,165)
FPKM
5
0
TSS
1kbp
Fosslie Xu Fosslie Mei Fosslie Xu Fosslie Xu Fosslie Liu Fosslie Liu Fosslie Liu
d
Fosslie et al.
P7 P10 P12 NSN SN MII Zygote 2–cell 4–cell 8–cell Morula Blast.
P7 0.66 0.46 0.51 0.46 0.53 0.47 0.43 0.32 0.36 0.40 0.41
P10 0.61 0.59 0.56 0.54 0.51 0.43 0.35 0.37 0.42 0.41
P12 0.73 0.70 0.59 0.57 0.48 0.46 0.54 0.56 0.56
NSN 0.81 0.78 0.69 0.52 0.48 0.56 0.60 0.61
SN 0.70 0.67 0.51 0.46 0.53 0.57 0.57
MII 0.71 0.48 0.41 0.46 0.53 0.53
Zygote 0.46 0.41 0.47 0.52 0.52
2–cell 0.47 0.54 0.53 0.56
4–cell 0.68 0.61 0.65
8–cell 0.78 0.87
Morula 0.83
Blast.
Spearman correlation
1 0.8 0.6 0.4 0.2 0
e
TSS
Promoter Flanks
Bivalent Promoters
Active Enhancer
PolyComb Repr. & Open Chromatin
Open Chromatin
Transcription & Exons
Weak Enhancer
Transcribed Enhancers
PolyComb Repressed
ZNF genes & ZNF Repeats
Transcription
Quiescent
Weak transcription
Heterochromatin
Assembly Gaps & Artifacts
-3 -1 1 3 5
Log2 relative enrichment (obs/exp)

Supplementary Fig. 2 | Oocyte and embryo H2A.Z signal is highly consistent with other published studies

a,**b,** Correlation matrices of H2A.Z enrichment throughout the whole genome, measured by FPKM value in 10 kb genomic bins. Pairwise for each study, the left plot shows correlations between samples from the same study whereas the right plot correlations between samples from different studies. **c**, Heatmaps of H2A.Z ChIP-seq signal in developmental stages in common with Xu et al.[27] and Mei et al.[28] at unique TSSs ±1kbp (n=32,166). Clustering was based on the distribution of all visualized H2A.Z signals. Blast., blastocyst. **d**, Correlation matrix of H2A.Z enrichment throughout the whole genome by the FPKM value in 10 kb genomic bins. **e**, Analysis of H2A.Z peaks in functional chromatin regions conserved across mouse tissues[30]. The average relative enrichment is shown with standard deviation as error bars. Repr., repressed.

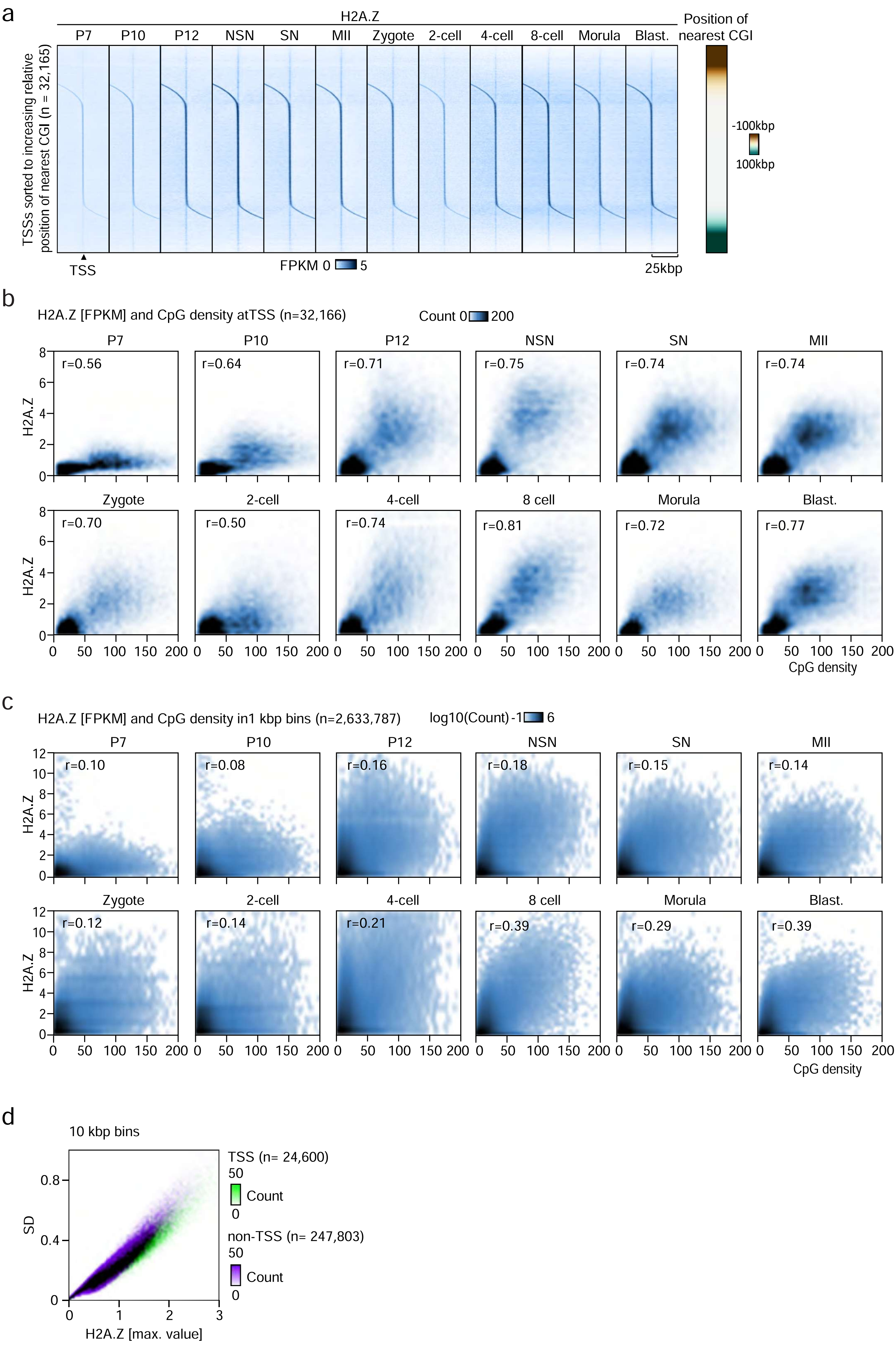
a
H2A.Z
P7
P10
P12
NSN
SN
MII
Zygote
2-cell
4-cell
8-cell
Morula
Blast.
Position of nearest CGI
-100kbp
100kbp
TSSs sorted to increasing relative position of nearest CGI (n = 32,165)
TSS
FPKM 0 5
25kbp
b
H2A.Z [FPKM] and CpG density atTSS (n=32,166)
Count 0 200
P7 r=0.56
P10 r=0.64
P12 r=0.71
NSN r=0.75
SN r=0.74
MII r=0.74
Zygote r=0.70
2-cell r=0.50
4-cell r=0.74
8 cell r=0.81
Morula r=0.72
Blast. r=0.77
H2A.Z
CpG density
c
H2A.Z [FPKM] and CpG density in1 kbp bins (n=2,633,787)
log10(Count) -1 6
P7 r=0.10
P10 r=0.08
P12 r=0.16
NSN r=0.18
SN r=0.15
MII r=0.14
Zygote r=0.12
2-cell r=0.14
4-cell r=0.21
8 cell r=0.39
Morula r=0.29
Blast. r=0.39
H2A.Z
CpG density
d
10 kbp bins
TSS (n= 24,600)
50 Count 0
non-TSS (n= 247,803)
50 Count 0
SD
H2A.Z [max. value]

Supplementary Fig. 3 | H2A.Z is correlated to CpG density at TSSs but not genome-wide
a, Heatmaps of H2A.Z signal in different developmental stages at unique TSSs ±25kbp (n=32,166) sorted according to increasing relative position of the nearest CGI. Blast., blastocyst. **b**, **c**, 2D-histograms showing the relationship between H2A.Z and CpG density in different developmental stages at (**b**) unique TSSs (n=32,166) ±1kbp and (**c**) throughout the whole genome (1kbp bins, n=2,633,787). r: Spearman's rank correlation coefficients; Blast., blastocyst. **d**, Overlaid 2D-histograms showing genome-wide relationships between maximum H2A.Z levels (X-axis) as well as standard deviations (Y-axis) of all stages analyzed in 10 kbp bins (n=263,389). Purple and green coloring represents bins that do and do not overlap with a TSS, respectively. Black coloring represents the overlay between these two populations.

Fig. S4

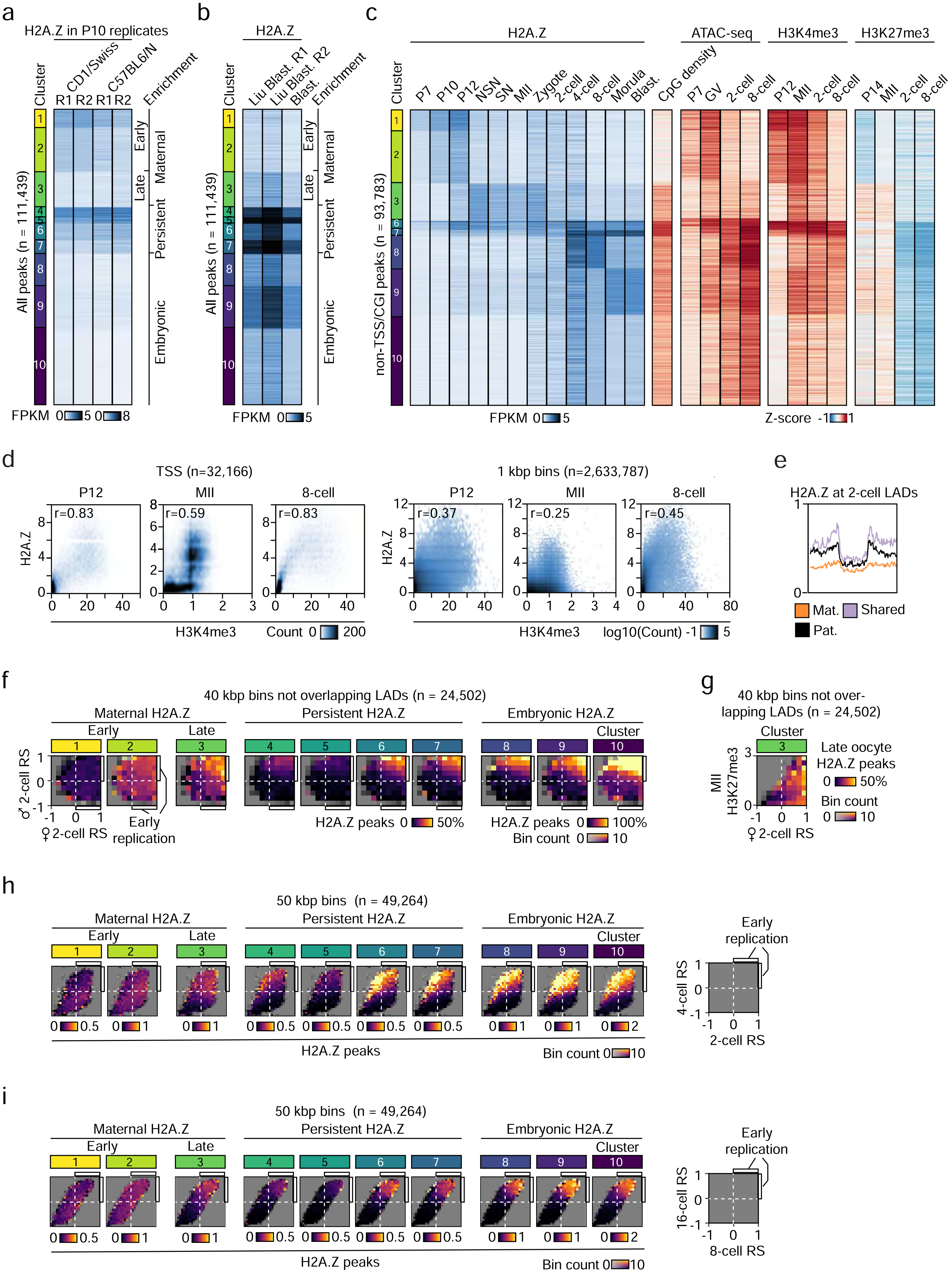
a
H2A.Z in P10 replicates
CD1/Swiss
C57BL6/N
R1 R2 R1 R2
Enrichment
Cluster
All peaks (n = 111,439)
Early
Late
Maternal
Persistent
Embryonic
FPKM 0 5 0 8
b
H2A.Z
Liu Blast. R1
Liu Blast. R2
Blast.
Enrichment
FPKM 0 5
c
H2A.Z
ATAC-seq
H3K4me3
H3K27me3
P7 P10 P12 NSN SN MII Zygote 2-cell 4-cell 8-cell Morula Blast.
CpG density
P7 GV 2-cell 8-cell
P12 MII 2-cell 8-cell
P14 MII 2-cell 8-cell
non-TSS/CGI peaks (n = 93,783)
FPKM 0 5
Z-score -1 1
d
TSS (n=32,166)
1 kbp bins (n=2,633,787)
P12 MII 8-cell
r=0.83 r=0.59 r=0.83
r=0.37 r=0.25 r=0.45
H2A.Z
H3K4me3
Count 0 200
log10(Count) -1 5
e
H2A.Z at 2-cell LADs
Mat. Shared
Pat.
f
40 kbp bins not overlapping LADs (n = 24,502)
Maternal H2A.Z
Persistent H2A.Z
Embryonic H2A.Z
Early
Late
Cluster
♂ 2-cell RS
♀ 2-cell RS
Early replication
H2A.Z peaks 0 50%
H2A.Z peaks 0 100%
Bin count 0 10
g
40 kbp bins not overlapping LADs (n = 24,502)
Cluster
MII H3K27me3
Late oocyte H2A.Z peaks 0 50%
Bin count 0 10
♀ 2-cell RS
h
50 kbp bins (n = 49,264)
Maternal H2A.Z
Persistent H2A.Z
Embryonic H2A.Z
H2A.Z peaks
Bin count 0 10
Early replication
4-cell RS
2-cell RS
i
50 kbp bins (n = 49,264)
H2A.Z peaks
Bin count 0 10
Early replication
16-cell RS
8-cell RS

Supplementary Fig. 4 | H2A.Z signal intensity is intricately associated to H3K4me3 levels, LADs, and replication timing

a, Heatmaps of H2A.Z enrichment at aggregated and clustered H2A.Z peaks at 4 biological replicates of P10 from two different mouse strains used in this study. **b**, Heatmaps of H2A.Z enrichment at aggregated and clustered H2A.Z peaks in blastocyst (blast.) replicates from[15] compared to blastocyst from this study. **c**, Heatmaps of H2A.Z enrichment at aggregated and clustered H2A.Z peaks during different developmental stages as well as Z-score of CpG density, histone marks[35,36,71] and ATAC-seq[37-39] in clustered peaks (non-TSS/CGI, n=93,783) compared to genome-wide signal. Blast., blastocyst. **d**, 2D-histograms showing the relationship between H2A.Z and H3K4me3[35] signal at selected stages at either TSSs (n=32,166) ±1kbp or whole genome (1kbp bins, n=2,633,787). r: Spearman's rank correlation coefficients. **e**, Graph of average peak density at and around different categories of LADs found in the paternal and maternal genomes of 2-cell embryos[22]. Horizontal bar illustrates the LAD location. **f**, 2D-histograms showing the genome-wide occurrence of each cluster of H2A.Z peaks (color) in relation to the mean Replication Status (RS) of the maternal (X-axis) and paternal (Y-axis) genomes[25] in individual 2-cell embryos. # and * indicates noteworthy sex-specific differences, where low and high peak-occurrences, respectively, largely follows the maternal RS, but not the paternal. Data was analyzed in 40 kbp bins, and the subset of bins not overlapping with LADs are shown (n=24,502). For the overlapping subset see Fig. 3d. **g**, 2D-histogram showing the genome-wide occurrence of cluster 3 H2A.Z peaks (color) in relation to the mean maternal RS (X-axis) in individual 2-cell embryos and H3K27me3 levels in MII oocytes (Y-axis). Data was analyzed in 40 kbp bins, and the subset of bins not overlapping with LADs are shown (n=24,502). For the overlapping subset see Fig. 3e. **h**, 2D-histograms showing the genome-wide occurrence of each cluster of H2A.Z peaks (color) in relation to the 2-cell (X-axis) and 4-cell (Y-axis) RS[42]. Data was analyzed in 50 kbp bins. **i**, 2D-histograms showing the genome-wide occurrence of each cluster of H2A.Z peaks (color) in relation to the 8-cell (X-axis) and 16-cell (Y-axis) RS[42]. Data was analyzed in 50 kbp bins.

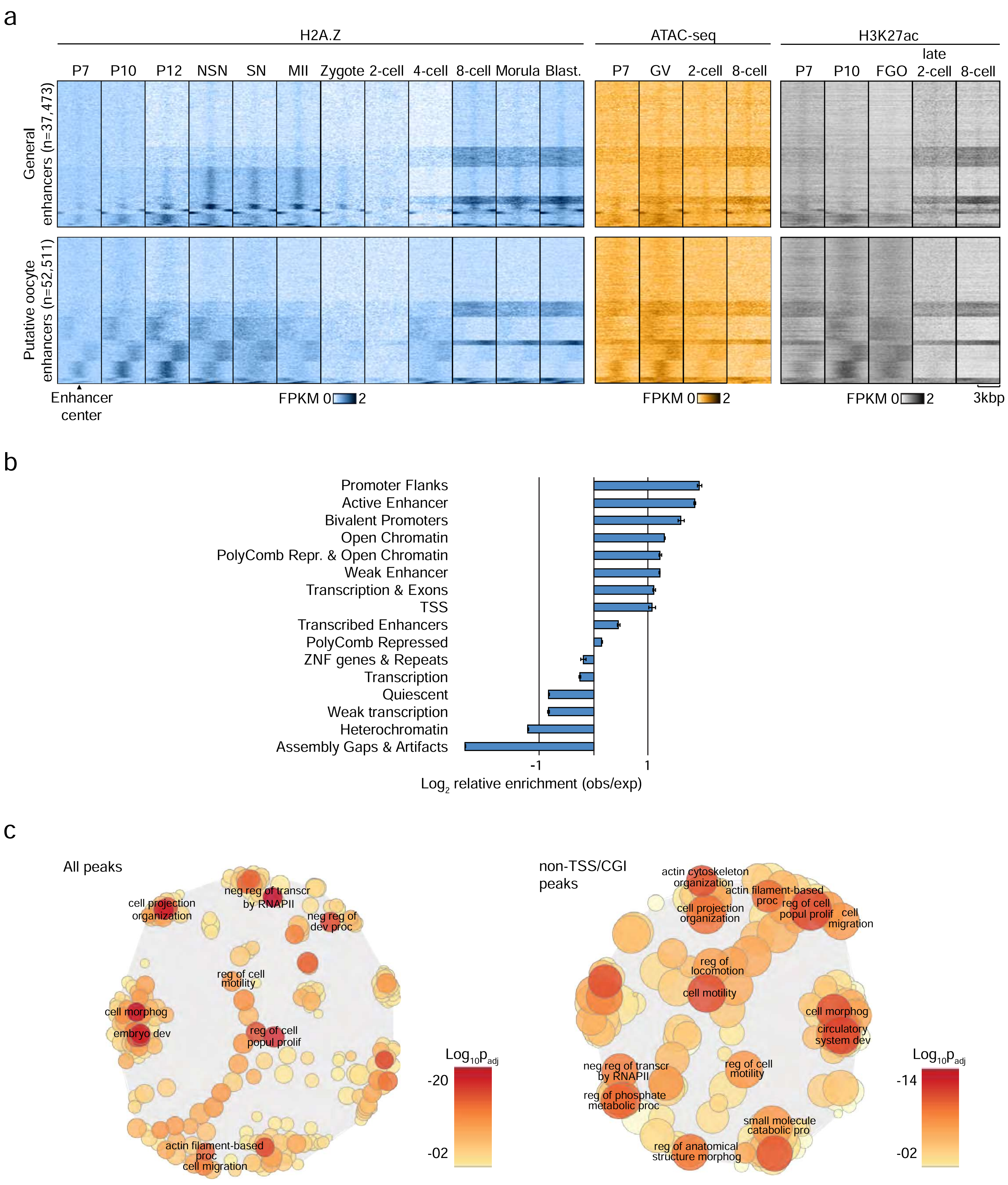
a
H2A.Z
ATAC-seq
H3K27ac
P7
P10
P12
NSN
SN
MII
Zygote
2-cell
4-cell
8-cell
Morula
Blast.
P7
GV
2-cell
8-cell
P7
P10
FGO
late 2-cell
8-cell
General enhancers (n=37,473)
Putative oocyte enhancers (n=52,511)
Enhancer center
FPKM 0 2
FPKM 0 2
FPKM 0 2
3kbp
b
Promoter Flanks
Active Enhancer
Bivalent Promoters
Open Chromatin
PolyComb Repr. & Open Chromatin
Weak Enhancer
Transcription & Exons
TSS
Transcribed Enhancers
PolyComb Repressed
ZNF genes & Repeats
Transcription
Quiescent
Weak transcription
Heterochromatin
Assembly Gaps & Artifacts
-1
1
Log2 relative enrichment (obs/exp)
c
All peaks
cell projection organization
neg reg of transcr by RNAPII
neg reg of dev proc
reg of cell motility
cell morphog
embryo dev
reg of cell popul prolif
actin filament-based proc
cell migration
Log10Padj
-20
-02
non-TSS/CGI peaks
actin cytoskeleton organization
actin filament-based proc
reg of cell popul prolif
cell migration
cell projection organization
reg of locomotion
cell motility
cell morphog
circulatory system dev
neg reg of transcr by RNAPII
reg of cell motility
reg of phosphate metabolic proc
small molecule catabolic pro
reg of anatomical structure morphog
Log10Padj
-14
-02

Supplementary Fig. 5 | H2A.Z signal on enhancers in oocytes and early embryos

a, H2A.Z signal at general enhancers (n=37,473)[45] and putative oocyte enhancers (n=52,511)[39] in different developmental stages, clustered based on the stage-specific distribution of the H2A.Z signal at and around enhancers. ATAC-seq[37-39] and H3K27ac[39] enrichment is visualized in the same clustering. Blast., blastocyst. **b**, Analysis of H2A.Z non-TSS/CGI peaks in functional chromatin regions conserved across mouse tissues[30]. The average relative enrichment is shown with standard deviation as error bars. Repr., repressed. **c**, Networks of enriched GO terms in all and non-TSS/CGI H2A.Z peak sets show significant enrichment in genes involved in transcriptional regulation, cell morphogenesis, and developmental processes. H2A.Z peaks were assigned to gene sets, the enriched processes (FDR $\leq$ 0.05) summarized by REVIGO[96], and plotted in Cytoscape. Exemplary GO terms for each cluster are annotated. Semantic similarity reflected in the clustering, color and font size reflect the $\log_{10}$ *FDR* value. Circle radius reflects the log of number of genes in GO term ID. dev, development; morphog, morphogenesis; neg, negative; popul, population; proc, processes; prolif, proliferation; reg, regulation; transcr, transcription.

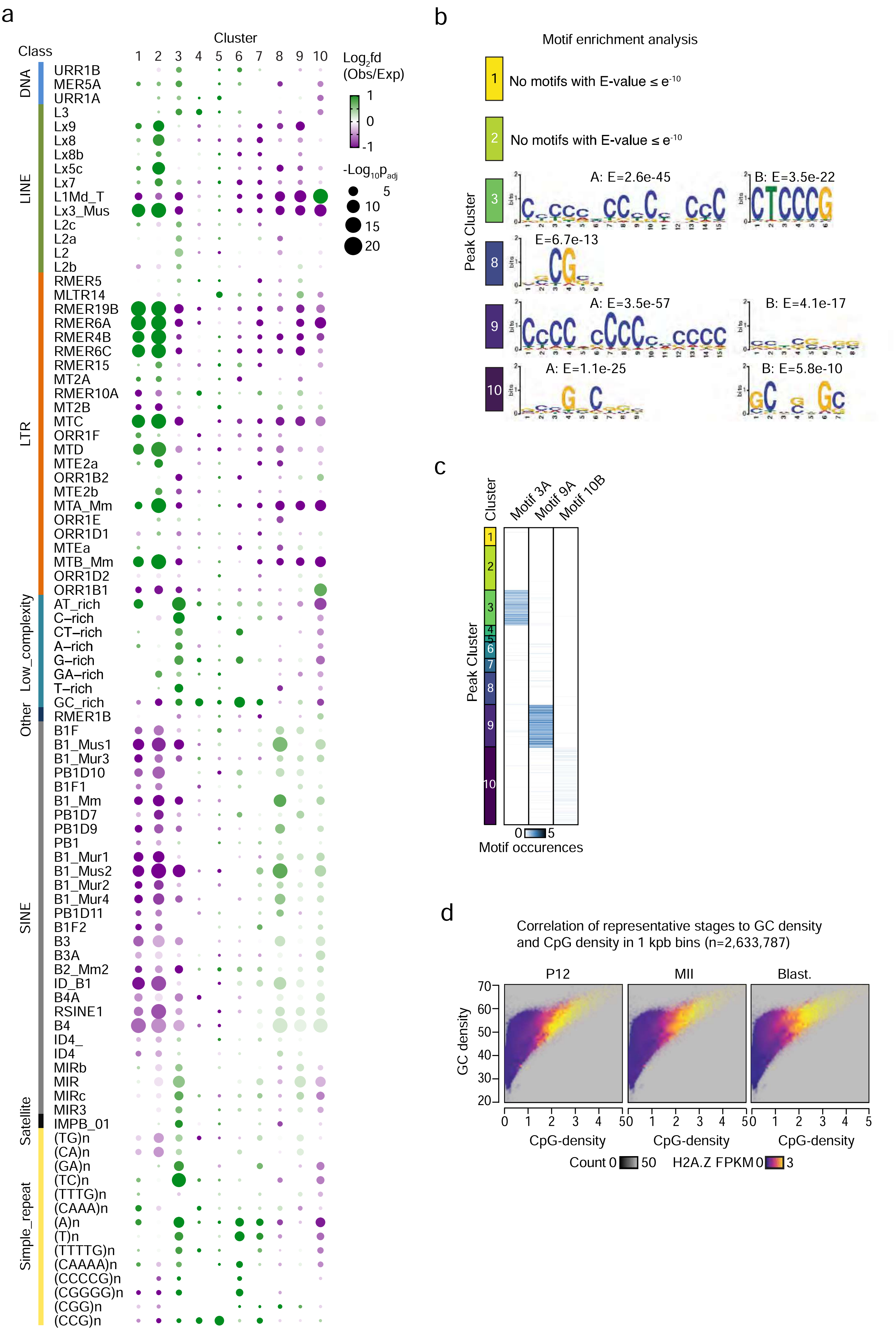
a
Cluster
Class
1 2 3 4 5 6 7 8 9 10
Log2fd (Obs/Exp)
1
0
-1
-Log10padj
5
10
15
20
DNA
URR1B
MER5A
URR1A
LINE
L3
Lx9
Lx8
Lx8b
Lx5c
Lx7
L1Md_T
Lx3_Mus
L2c
L2a
L2
L2b
LTR
RMER5
MLTR14
RMER19B
RMER6A
RMER4B
RMER6C
RMER15
MT2A
RMER10A
MT2B
MTC
ORR1F
MTD
MTE2a
ORR1B2
MTE2b
MTA_Mm
ORR1E
ORR1D1
MTEa
MTB_Mm
ORR1D2
ORR1B1
Low_complexity
AT_rich
C–rich
CT–rich
A–rich
G–rich
GA–rich
T–rich
GC_rich
Other
RMER1B
SINE
B1F
B1_Mus1
B1_Mur3
PB1D10
B1F1
B1_Mm
PB1D7
PB1D9
PB1
B1_Mur1
B1_Mus2
B1_Mur2
B1_Mur4
PB1D11
B1F2
B3
B3A
B2_Mm2
ID_B1
B4A
RSINE1
B4
ID4_
ID4
MIRb
MIR
MIRc
MIR3
Satellite
IMPB_01
Simple_repeat
(TG)n
(CA)n
(GA)n
(TC)n
(TTTG)n
(CAAA)n
(A)n
(T)n
(TTTTG)n
(CAAAA)n
(CCCCG)n
(CGGGG)n
(CGG)n
(CCG)n
b
Motif enrichment analysis
1 No motifs with E-value ≤ e-10
2 No motifs with E-value ≤ e-10
Peak Cluster
3 A: E=2.6e-45
B: E=3.5e-22
8 E=6.7e-13
9 A: E=3.5e-57
B: E=4.1e-17
10 A: E=1.1e-25
B: E=5.8e-10
c
Cluster
Motif 3A
Motif 9A
Motif 10B
Peak Cluster
1 2 3 4 5 6 7 8 9 10
0 5
Motif occurences
d
Correlation of representative stages to GC density
and CpG density in 1 kpb bins (n=2,633,787)
P12
MII
Blast.
GC density
70
60
50
40
30
20
0 1 2 3 4 5
CpG-density
Count 0 50
H2A.Z FPKM 0 3

Supplementary Fig. 6 | H2A.Z signal is correlated to CpG density and found at specific RTs

a, Bubble plots showing overlap of repetitive element subtypes in clusters of non-TSS/CGI H2A.Z peaks compared to an average distribution across all clusters. *p*-values were calculated by Chi Square tests Benjamini-Hochberg adjusted for multiple testing. **b**, Graphical presentations of nucleotide composition and frequencies in the most enriched motifs at selected clusters of aggregate H2A.Z peaks found using MEME-ChIP[98]. Peaks were identified and clustered as Fig. 3a, and peaks from oocyte specific clusters (1, 2 and 3) and embryo specific clusters (8,9 and 10) not overlapping with TSSs or CGIs were tested. Control regions were based on distance to the nearest TSS, and only hits with E-value $\leq e^{-10}$ are shown. **c**, Selected motif occurrences from b shown at aggregated and clustered H2A.Z peaks. **d,** 2D-histograms showing the relationship between GC density, CpG density and H2A.Z signal from selected developmental stages throughout the whole genome (1 kbp bins, n=2,633,787). Blast., blastocyst.

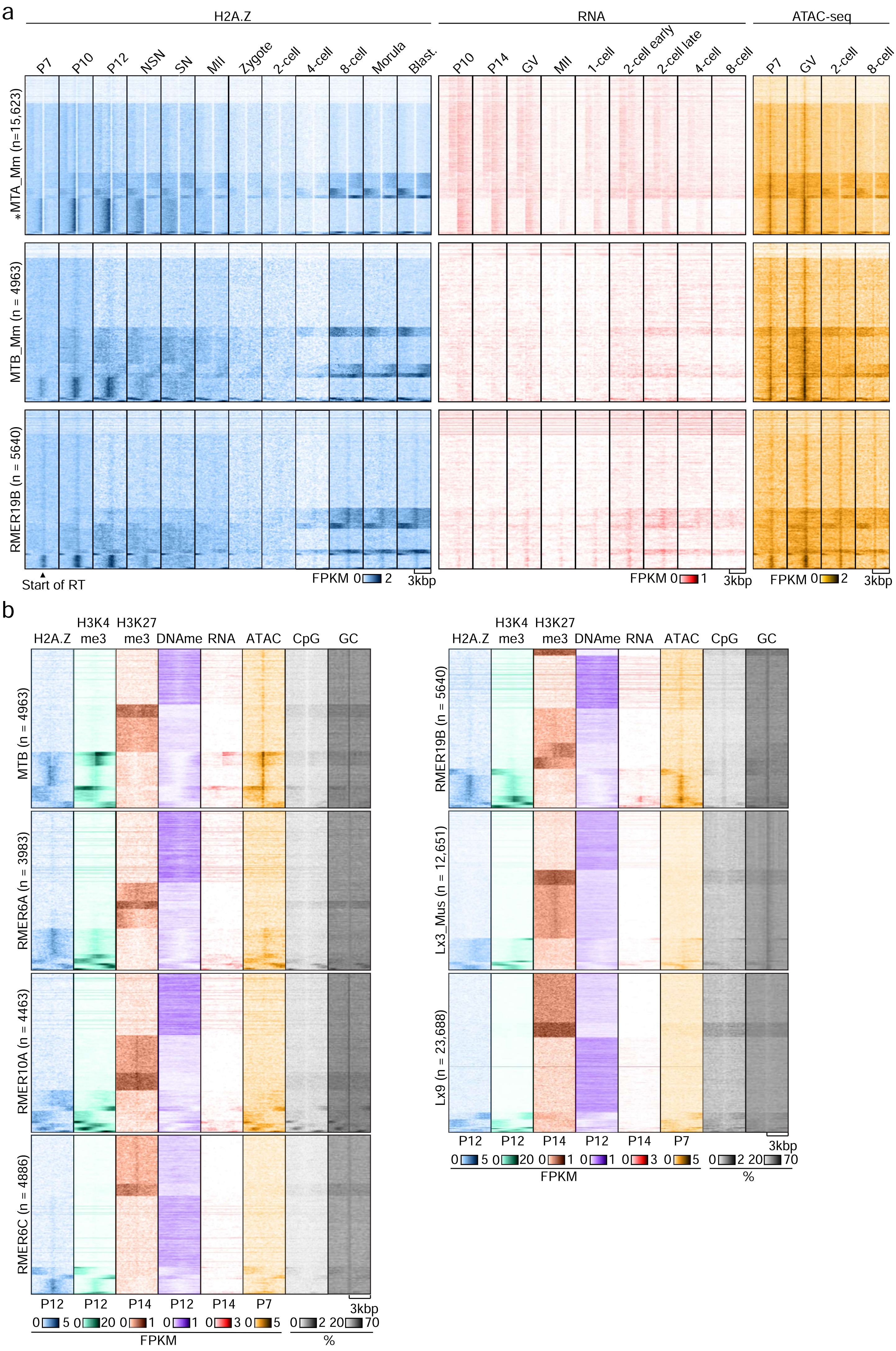
a
H2A.Z
RNA
ATAC-seq
P7
P10
P12
NSN
SN
MII
Zygote
2-cell
4-cell
8-cell
Morula
Blast.
P10
P14
GV
MII
1-cell
2-cell early
2-cell late
4-cell
8-cell
P7
GV
2-cell
8-cell
*MTA_Mm (n=15,623)
MTB_Mm (n = 4963)
RMER19B (n = 5640)
Start of RT
FPKM 0 2
3kbp
FPKM 0 1
3kbp
FPKM 0 2
3kbp
b
H2A.Z
H3K4 me3
H3K27 me3
DNAme
RNA
ATAC
CpG
GC
MTB (n = 4963)
RMER6A (n = 3983)
RMER10A (n = 4463)
RMER6C (n = 4886)
P12
P12
P14
P12
P14
P7
3kbp
0 5 0 20 0 1 0 1 0 3 0 5
0 2 20 70
FPKM
%
H2A.Z
H3K4 me3
H3K27 me3
DNAme
RNA
ATAC
CpG
GC
RMER19B (n = 5640)
Lx3_Mus (n = 12,651)
Lx9 (n = 23,688)
P12
P12
P14
P12
P14
P7
3kbp
0 5 0 20 0 1 0 1 0 3 0 5
0 2 20 70
FPKM
%

Supplementary Fig. 7 | H2A.Z peaks overlap with specific repetitive elements
a, Heatmaps of H2A.Z, ATAC-seq[37-39] and RNA signal[31] surrounding the start of MTA, MTB and RMER19B LTR RTs in different developmental stages clustered based on H2A.Z in all stages. * Highlights cluster with the strongest H2A.Z signal in P12 oocytes. Blast., blastocyst.
b, Heatmaps showing indicated features[31,39,72] at selected RTs clustered based on the combined distribution of H2A.Z, H3K4me3[35] and H3K27me3[71] at the start of each RT ±3kbp in growing oocytes (P12 or P14).
