# Supplementary material for: Major waves of H2A.Z incorporation during mouse oogenesis and preimplantation embryo development
Source: Nat Commun. 2025 Dec 2;17:210. doi: 10.1038/s41467-025-66919-x (PMC12779981; doi:10.1038/s41467-025-66919-x)
Supplement: Supplementary file 2 — Description of Additional Supplementary Files [file 41467_2025_66919_MOESM2_ESM.pdf]

Description of Additional Supplementary Files

File name: Supplementary Data 1

Description: NGS processing summary of H2A.Z picoChIP samples.

File name: Supplementary Data 2

Description: List of all H2A.Z peaks identified and clustered in oocytes and early embryos excluding zygote, 2-cell and 4-cell stages.

File name: Supplementary Data 3

Description: Gene ontology enrichment analysis of H2A.Z peak clusters. One-tailed Fisher's exact test was used to determine the p-values. Multiple testing correction was done using g:SCS.

File name: Supplementary Data 4

Description: Collected mouse oocyte and early embryo sample pools for H2A.Z picoChIP..

File name: Supplementary Data 5

Description: Antibody information for H2A.Z picoChIP.

File name: Supplementary Data 6

Description: Sonication parameters for picoChIP.

File name: Supplementary Data 7

Description: Sonicated chromatin preparation for H2A.Z picoChIP.

File name: Supplementary Data 8

Description: Sample washing steps for H2A.Z picoChIP.

File name: Supplementary Data 9

Description: Underlying data for bubble plots showing overlaps between TSS-associated H2A.Z

peaks and oocyte/embryonic gene categories, all H2A.Z peaks and LADs, and non-TSS/CGI H2A.Z peaks and TEs compared to an average distribution across all clusters. p-values were calculated by Chi Square tests and adjusted for multiple testing by Benjamini-Hochberg.
