# Supplementary material for: Major waves of H2A.Z incorporation during mouse oogenesis and preimplantation embryo development
Source: Nat Commun. 2025 Dec 2;17:210. doi: 10.1038/s41467-025-66919-x (PMC12779981; doi:10.1038/s41467-025-66919-x)
Supplement: Supplementary file 12 — Reporting Summary [file 41467_2025_66919_MOESM12_ESM.pdf]

nature portfolio

Corresponding author(s): Mika Zagrobelny, John Arne Dahl, Mads Lerdrup

Last updated by author(s): Apr 11, 2025

Reporting Summary

Nature Portfolio wishes to improve the reproducibility of the work that we publish. This form provides structure for consistency and transparency in reporting. For further information on Nature Portfolio policies, see our Editorial Policies and the Editorial Policy Checklist.

Statistics

For all statistical analyses, confirm that the following items are present in the figure legend, table legend, main text, or Methods section.

n/a	Confirmed	
☐	☒	The exact sample size (*n*) for each experimental group/condition, given as a discrete number and unit of measurement
☐	☒	A statement on whether measurements were taken from distinct samples or whether the same sample was measured repeatedly
☐	☒	The statistical test(s) used AND whether they are one- or two-sided<br>*Only common tests should be described solely by name; describe more complex techniques in the Methods section.*
☐	☒	A description of all covariates tested
☐	☒	A description of any assumptions or corrections, such as tests of normality and adjustment for multiple comparisons
☐	☒	A full description of the statistical parameters including central tendency (e.g. means) or other basic estimates (e.g. regression coefficient) AND variation (e.g. standard deviation) or associated estimates of uncertainty (e.g. confidence intervals)
☐	☒	For null hypothesis testing, the test statistic (e.g. F, t, r) with confidence intervals, effect sizes, degrees of freedom and P value noted<br>*Give P values as exact values whenever suitable.*
☒	☐	For Bayesian analysis, information on the choice of priors and Markov chain Monte Carlo settings
☒	☐	For hierarchical and complex designs, identification of the appropriate level for tests and full reporting of outcomes
☒	☐	Estimates of effect sizes (e.g. Cohen's *d*, Pearson's *r*), indicating how they were calculated

Our web collection on statistics for biologists contains articles on many of the points above.

Software and code

Policy information about availability of computer code

Data collection	Data collection from GEO database was done using scripts that can be found in GitHub repository: the https://github.com/lerdruplab/sra_scripts. In detail, GSM numbers for data of interest were used to download GEO metadata and SRR accession list from SRA Run Selector website (https://www.ncbi.nlm.nih.gov/Traces/study/). 01_get_samplenames_table.R script was used to generate a table containing SRR numbers and matching sample names. SRR accession list downloaded from SRA Run Selector was then used to prefetch .sra files using sra-tools v3.1.1. 02_fastq_dumb.sh script was used to get fastq files using sra-tools v3.1.1. 03_replace_samplenames.sh script was used to rename the fastq files accordingly based on the table output of 01_get_samplenames_table.R script.
Data analysis	R scripts used for analysis and visualization can be found in the Github repositoy: https://github.com/lerdruplab/H2A.Z.

For manuscripts utilizing custom algorithms or software that are central to the research but not yet described in published literature, software must be made available to editors and reviewers. We strongly encourage code deposition in a community repository (e.g. GitHub). See the Nature Portfolio guidelines for submitting code & software for further information.

Data

Policy information about availability of data

All manuscripts must include a data availability statement. This statement should provide the following information, where applicable:

- Accession codes, unique identifiers, or web links for publicly available datasets
- A description of any restrictions on data availability
- For clinical datasets or third party data, please ensure that the statement adheres to our policy

Data availability
All new ChIP-seq data are deposited at NCBI's Gene Expression Omnibus (Edgar, Domrachev, & Lash, 2002) under the accession number GSE293415.
------ FOR REVIEWERS (will be deleted from the manuscript upon acceptance): ------
To review GEO accession GSE293415:
Reviewer access to the unpublished ChIP-seq data in this study can be obtained using the link https://www.ncbi.nlm.nih.gov/geo/query/acc.cgi?acc=GSE293415 and by entering the token qncfsqiwthgntqj into the box.

Research involving human participants, their data, or biological material

Policy information about studies with human participants or human data. See also policy information about sex, gender (identity/presentation), and sexual orientation and race, ethnicity and racism.

Reporting on sex and gender	Not relevant
Reporting on race, ethnicity, or other socially relevant groupings	Not relevant
Population characteristics	Not relevant
Recruitment	Not relevant
Ethics oversight	Not relevant

Note that full information on the approval of the study protocol must also be provided in the manuscript.

Field-specific reporting

Please select the one below that is the best fit for your research. If you are not sure, read the appropriate sections before making your selection.

- [x] Life sciences
- [ ] Behavioural & social sciences
- [ ] Ecological, evolutionary & environmental sciences

For a reference copy of the document with all sections, see nature.com/documents/nr-reporting-summary-flat.pdf

Life sciences study design

All studies must disclose on these points even when the disclosure is negative.

Sample size	The number of cells used for samples are listed in Table 1 in the methods. In general oocyte and embryo numbers were decided to balance signal strength and limit the number of sacrificed animals.
Data exclusions	No data were excluded
Replication	Replicate samples were generated and compared for the P10 stage. Data were subject to thorough cross-validation to previously published ES cell data and blastocyst data shown in Figure S1.
Randomization	No scoring requiring randomization was done.
Blinding	No scoring requiring blinding was done.

Reporting for specific materials, systems and methods

We require information from authors about some types of materials, experimental systems and methods used in many studies. Here, indicate whether each material, system or method listed is relevant to your study. If you are not sure if a list item applies to your research, read the appropriate section before selecting a response.

Materials & experimental systems

n/a	Involved in the study	
☐	☒	Antibodies
☐	☒	Eukaryotic cell lines
☒	☐	Palaeontology and archaeology
☐	☒	Animals and other organisms
☒	☐	Clinical data
☒	☐	Dual use research of concern
☒	☐	Plants

Methods

n/a	Involved in the study	
☐	☒	ChIP-seq
☒	☐	Flow cytometry
☒	☐	MRI-based neuroimaging

Antibodies

Antibodies used

H2A.Z cat# 39114, Active Motif Lot. 06217001

Validation

Antibody amounts are provided in the methods.
- For ChIP, this is in table 2. ("Clone name" is not relevant since this is a polyclonal antibody).
- For immunostaining, it is described as "Embryos were incubated in blocking solution with 1:1000 α-H2A.Z antibody (Table 2) for 1 h at room temperature".

Applications Validated by Active Motif (https://www.activemotif.com/catalog/details/39113.html):
ChIP-Seq: 5 μl each
WB*: 1:1,000 - 1:5,000 dilution

Our generated data were subject to thorough cross-validation to previously published ES cell data as well as recently published data from oocytes and embryos in Supplementary Figures 1+2.

Eukaryotic cell lines

Policy information about cell lines and Sex and Gender in Research

Cell line source(s)

State the source of each cell line used and the sex of all primary cell lines and cells derived from human participants or vertebrate models.

Authentication

Describe the authentication procedures for each cell line used OR declare that none of the cell lines used were authenticated.

Mycoplasma contamination

Confirm that all cell lines tested negative for mycoplasma contamination OR describe the results of the testing for mycoplasma contamination OR declare that the cell lines were not tested for mycoplasma contamination.

Commonly misidentified lines
(See ICLAC register)

Name any commonly misidentified cell lines used in the study and provide a rationale for their use.

Animals and other research organisms

Policy information about studies involving animals; ARRIVE guidelines recommended for reporting animal research, and Sex and Gender in Research

Laboratory animals

This information is also provided in the manuscript:
- Mouse strain source: C57BL/6NRj mice (Black 6N, Janvier Labs, France) and RjOrl:SWISS mice (CD-1®, Janvier Labs)
- Substrain: Black 6 mice are of the N-type, CD-1 mice are of the SWISS type, also mentioned in the naming above.
- Genetic background: These are wildtype mice
- Species: Mice
- Age of animals: Postnatal day 7 (P7), P10, and P12 CD-1 females were used for collection of growing oocyte (GO) sample pools, as well as P10 Black 6N females. Black 6N females aged 4 weeks were used for collecting NSN, SN and MII oocytes (Table 1)

Wild animals

Not applicable

Reporting on sex

The study involves gender specific information for gametes (oocytes). Embryos were not analyzed or selected for gender, so we expect an equal mix of male and females.

Field-collected samples

Not applicable

Ethics oversight

All mouse experiments were approved and registered by the Norwegian Food Safety Authority (NFSA approved application/FOTS IDs: 7216, 10898, and 24911, and 8743) and conducted in accordance with Norwegian regulation FOR-2015-06-18-761, which closely aligns with EU directive 2010/63/EU on stringent ethical and welfare standards to protect animals used for scientific purposes.

Note that full information on the approval of the study protocol must also be provided in the manuscript.

Plants

Seed stocks

Report on the source of all seed stocks or other plant material used. If applicable, state the seed stock centre and catalogue number. If plant specimens were collected from the field, describe the collection location, date and sampling procedures.

Novel plant genotypes

Describe the methods by which all novel plant genotypes were produced. This includes those generated by transgenic approaches, gene editing, chemical/radiation-based mutagenesis and hybridization. For transgenic lines, describe the transformation method, the number of independent lines analyzed and the generation upon which experiments were performed. For gene-edited lines, describe the editor used, the endogenous sequence targeted for editing, the targeting guide RNA sequence (if applicable) and how the editor was applied.

Authentication

Describe any authentication procedures for each seed stock used or novel genotype generated. Describe any experiments used to assess the effect of a mutation and, where applicable, how potential secondary effects (e.g. second site T-DNA insertions, mosiacism, off-target gene editing) were examined.

ChIP-seq

Data deposition

☒ Confirm that both raw and final processed data have been deposited in a public database such as GEO.

☒ Confirm that you have deposited or provided access to graph files (e.g. BED files) for the called peaks.

Data access links
May remain private before publication.

To review GEO accession GSE293415:
Reviewer access to the unpublished ChIP-seq data in this study can be obtained using the link https://www.ncbi.nlm.nih.gov/geo/query/acc.cgi?acc=GSE293415 and by entering the token qncfsqiwthgntqj into the box.

Files in database submission

1-100kAZstd_S1_R1_001.fastq.gz
1-100kAZstd_S1.wig.gz
1-p10A-AZ-250-11_S8_R1_001.fastq.gz
1-p10A-AZ-250-11_S8_R1_001.wig.gz
1-p10A-AZ-250-11_S8_R2_001.fastq.gz
1-p12-AZ-200_S1_L001_R1_001.fastq.gz
1-p12-AZ-200_S1_L001_R1_001.wig.gz
1-p12-AZ-200_S1_L001_R2_001.fastq.gz
1-p12-AZ-200_S1_L002_R1_001.fastq.gz
1-p12-AZ-200_S1_L002_R2_001.fastq.gz
1-p12-AZ-200-B1_S1_R1_001.fastq.gz
1-p12-AZ-200-B1_S1_R2_001.fastq.gz
2-10kAZstd_S2_R1_001.fastq.gz
2-10kAZstd_S2.wig.gz
3-NSN-AZ-200_S3_L001_R1_001.fastq.gz
3-NSN-AZ-200_S3_L001_R1_001.wig.gz
3-NSN-AZ-200_S3_L001_R2_001.fastq.gz
3-NSN-AZ-200_S3_L002_R1_001.fastq.gz
3-NSN-AZ-200_S3_L002_R2_001.fastq.gz
3-NSN-AZ-200-B3_S4_R1_001.fastq.gz
3-NSN-AZ-200-B3_S4_R2_001.fastq.gz
4-p10B-AZ-250-14_S10_R1_001.fastq.gz
4-p10B-AZ-250-14_S10_R1_001.wig.gz
4-p10B-AZ-250-14_S10_R2_001.fastq.gz
4-SN-AZ-200_S4_L001_R1_001.fastq.gz
4-SN-AZ-200_S4_L001_R1_001.wig.gz
4-SN-AZ-200_S4_L001_R2_001.fastq.gz
4-SN-AZ-200_S4_L002_R1_001.fastq.gz
4-SN-AZ-200_S4_L002_R2_001.fastq.gz
4-SN-AZ-200-B4_S20_R1_001.fastq.gz
4-SN-AZ-200-B4_S20_R2_001.fastq.gz
5-5-500-AZ-oc_S5_R1_001.fastq.gz
5-5-500-AZ-oc_S5.wig.gz
5-AZINP_S5_R1_001.fastq.gz
5-AZINP_S5.wig.gz
5-MII-AZ-300_S5_L001_R1_001.fastq.gz
5-MII-AZ-300_S5_L001_R1_001.wig.gz
5-MII-AZ-300_S5_L001_R2_001.fastq.gz
5-MII-AZ-300_S5_L002_R1_001.fastq.gz
5-MII-AZ-300_S5_L002_R2_001.fastq.gz
5-MII-AZ-300-B5_S7_R1_001.fastq.gz

5-MII-AZ-300-B5_S7_R2_001.fastq.gz
6-p7-Az-250-16_S14_R1_001.fastq.gz
6-p7-Az-250-16_S14_R1_001.wig.gz
6-p7-Az-250-16_S14_R2_001.fastq.gz
7-7-1k-AZ-oc_S7_R1_001.fastq.gz
7-7-1k-AZ-oc_S7.wig.gz
7-MII-33-50_S7_L001_R1_001.fastq.gz
7-MII-33-50_S7_L001_R2_001.fastq.gz
7-MII-33-50_S7_L002_R1_001.fastq.gz
7-MII-33-50_S7_L002_R2_001.fastq.gz
8-8c-AZ-460_S8_L001_R1_001.fastq.gz
8-8c-AZ-460_S8_L001_R1_001.wig.gz
8-8c-AZ-460_S8_L001_R2_001.fastq.gz
8-8c-AZ-460_S8_L002_R1_001.fastq.gz
8-8c-AZ-460_S8_L002_R2_001.fastq.gz
11-mor-AZ460_S11_L001_R1_001.fastq.gz
11-mor-AZ460_S11_L001_R1_001.wig.gz
11-mor-AZ460_S11_L001_R2_001.fastq.gz
11-mor-AZ460_S11_L002_R1_001.fastq.gz
11-mor-AZ460_S11_L002_R2_001.fastq.gz
11-p10A-INP-21_S2_R1_001.fastq.gz
11-p10A-INP-21_S2_R1_001.wig.gz
11-p10A-INP-21_S2_R2_001.fastq.gz
12-p10B-INP-22_S11_R1_001.fastq.gz
12-p10B-INP-22_S11_R1_001.wig.gz
12-p10B-INP-22_S11_R2_001.fastq.gz
13-bla-AZ460_S13_L001_R1_001.fastq.gz
13-bla-AZ460_S13_L001_R1_001.wig.gz
13-bla-AZ460_S13_L001_R2_001.fastq.gz
13-bla-AZ460_S13_L002_R1_001.fastq.gz
13-bla-AZ460_S13_L002_R2_001.fastq.gz
13-p7-INP-25_S12_R1_001.fastq.gz
13-p7-INP-25_S12_R1_001.wig.gz
13-p7-INP-25_S12_R2_001.fastq.gz
14-p12B-INP-27_S15_R1_001.fastq.gz
14-p12B-INP-27_S15_R1_001.wig.gz
14-p12B-INP-27_S15_R2_001.fastq.gz
15-INP-p12_S15_L001_R1_001.fastq.gz
15-INP-p12_S15_L001_R1_001.wig.gz
15-INP-p12_S15_L001_R2_001.fastq.gz
15-INP-p12_S15_L002_R1_001.fastq.gz
15-INP-p12_S15_L002_R2_001.fastq.gz
16-INP-NSN_S16_L001_R1_001.fastq.gz
16-INP-NSN_S16_L001_R1_001.wig.gz
16-INP-NSN_S16_L001_R2_001.fastq.gz
16-INP-NSN_S16_L002_R1_001.fastq.gz
16-INP-NSN_S16_L002_R2_001.fastq.gz
18-INP-SN_S17_L001_R1_001.fastq.gz
18-INP-SN_S17_L001_R1_001.wig.gz
18-INP-SN_S17_L001_R2_001.fastq.gz
18-INP-SN_S17_L002_R1_001.fastq.gz
18-INP-SN_S17_L002_R2_001.fastq.gz
19-INP-MII_S18_L001_R1_001.fastq.gz
19-INP-MII_S18_L001_R1_001.wig.gz
19-INP-MII_S18_L001_R2_001.fastq.gz
19-INP-MII_S18_L002_R1_001.fastq.gz
19-INP-MII_S18_L002_R2_001.fastq.gz
20-INP-8c_S19_L001_R1_001.fastq.gz
20-INP-8c_S19_L001_R1_001.wig.gz
20-INP-8c_S19_L001_R2_001.fastq.gz
20-INP-8c_S19_L002_R1_001.fastq.gz
20-INP-8c_S19_L002_R2_001.fastq.gz
21-INP-mor_S20_L001_R1_001.fastq.gz
21-INP-mor_S20_L001_R1_001.wig.gz
21-INP-mor_S20_L001_R2_001.fastq.gz
21-INP-mor_S20_L002_R1_001.fastq.gz
21-INP-mor_S20_L002_R2_001.fastq.gz
22-INP-bla_S21_L001_R1_001.fastq.gz
22-INP-bla_S21_L001_R1_001.wig.gz

22-INP-bla_S21_L001_R2_001.fastq.gz
22-INP-bla_S21_L002_R1_001.fastq.gz
22-INP-bla_S21_L002_R2_001.fastq.gz
1-MC-ChIP1-2AZ-p10-80-20250804_S1_L001_R1_001.fastq.gz
1-MC-ChIP1-2AZ-p10-80-20250804_S1_L001_R2_001.fastq.gz
1-MC-ChIP1-2AZ-p10-80-20250804_S1_L001_R1_001.wig.gz
2-MC-ChIP2-2AZ-p10-80-20250804_S2_L001_R1_001.fastq.gz
2-MC-ChIP2-2AZ-p10-80-20250804_S2_L001_R2_001.fastq.gz
2-MC-ChIP2-2AZ-p10-80-20250804_S2_L001_R1_001.wig.gz
3-MC-ChIP1-2AZ-Zyg-200-20250804_S3_L001_R1_001.fastq.gz
3-MC-ChIP1-2AZ-Zyg-200-20250804_S3_L001_R2_001.fastq.gz
3-MC-ChIP1-2AZ-Zyg-200-20250804_S3_L001_R1_001.wig.gz
4-MC-ChIP1-2AZ-2c-200-20250804_S4_L001_R1_001.fastq.gz
4-MC-ChIP1-2AZ-2c-200-20250804_S4_L001_R2_001.fastq.gz
4-MC-ChIP1-2AZ-2c-200-20250804_S4_L001_R1_001.wig.gz
5-MC-ChIP1-2AZ-4c-200-20250804_S5_L001_R1_001.fastq.gz
5-MC-ChIP1-2AZ-4c-200-20250804_S5_L001_R2_001.fastq.gz
5-MC-ChIP1-2AZ-4c-200-20250804_S5_L001_R1_001.wig.gz
6-MC-Input1-2AZ-p10-80-20250804_S6_L001_R1_001.fastq.gz
6-MC-Input1-2AZ-p10-80-20250804_S6_L001_R2_001.fastq.gz
6-MC-Input1-2AZ-p10-80-20250804_S6_L001_R1_001.wig.gz
7-MC-Input2-2AZ-p10-80-20250804_S7_L001_R1_001.fastq.gz
7-MC-Input2-2AZ-p10-80-20250804_S7_L001_R2_001.fastq.gz
7-MC-Input2-2AZ-p10-80-20250804_S7_L001_R1_001.wig.gz
8-MC-Input1-2AZ-Zyg-200-20250804_S8_L001_R1_001.fastq.gz
8-MC-Input1-2AZ-Zyg-200-20250804_S8_L001_R2_001.fastq.gz
8-MC-Input1-2AZ-Zyg-200-20250804_S8_L001_R1_001.wig.gz
9-MC-Input1-2AZ-2c-200-20250804_S9_L001_R1_001.fastq.gz
9-MC-Input1-2AZ-2c-200-20250804_S9_L001_R2_001.fastq.gz
9-MC-Input1-2AZ-2c-200-20250804_S9_L001_R1_001.wig.gz
10-MC-Input1-2AZ-4c-200-20250804_S10_L001_R1_001.fastq.gz
10-MC-Input1-2AZ-4c-200-20250804_S10_L001_R2_001.fastq.gz
10-MC-Input1-2AZ-4c-200-20250804_S10_L001_R1_001.wig.gz

Genome browser session
(e.g. UCSC)

Provide a link to an anonymized genome browser session for "Initial submission" and "Revised version" documents only, to enable peer review. Write "no longer applicable" for "Final submission" documents.

Methodology

Replicates

Replicate samples were generated and compared for the P10 stage. Data were subject to thorough cross-validation to previously published ES cell data and blastocyst data shown in Figure S1.

Sequencing depth

1-p10A-AZ-250-11_S8_R1_001, total number of reads: 50568561, uniquely mapped reads: 34488538, read length: 20-51, single-end
1-p12-AZ-200_S1_L001_R1_001, total number of reads: 30974415, uniquely mapped reads: 20081243, read length: 20-51, single-end
1-p12-AZ-200-B1_S1_R1_001, total number of reads: 9059520, uniquely mapped reads: 5970741, read length: 20-51, single-end
3-NSN-AZ-200_S3_L001_R1_001, total number of reads: 31740852, uniquely mapped reads: 23480890, read length: 20-51, single-end
3-NSN-AZ-200-B3_S4_R1_001, total number of reads: 20841255, uniquely mapped reads: 15519065, read length: 20-51, single-end
4-p10B-AZ-250-14_S10_R1_001, total number of reads: 36964044, uniquely mapped reads: 25535036, read length: 20-51, single-end
4-SN-AZ-200_S4_L001_R1_001, total number of reads: 39220514, uniquely mapped reads: 26999526, read length: 20-51, single-end
4-SN-AZ-200-B4_S20_R1_001, total number of reads: 6400973, uniquely mapped reads: 4507054, read length: 20-51, single-end
5-MII-AZ-300_S5_L001_R1_001, total number of reads: 32584186, uniquely mapped reads: 24704151, read length: 20-51, single-end
5-MII-AZ-300-B5_S7_R1_001, total number of reads: 7157557, uniquely mapped reads: 5436103, read length: 20-51, single-end
6-p7-Az-250-16_S14_R1_001, total number of reads: 40880500, uniquely mapped reads: 28650165, read length: 20-51, single-end
8-8c-AZ-460_S8_L001_R1_001, total number of reads: 38854867, uniquely mapped reads: 28437392, read length: 20-51, single-end
11-mor-AZ460_S11_L001_R1_001, total number of reads: 23502061, uniquely mapped reads: 16454864, read length: 20-51, single-end
11-p10A-INP-21_S2_R1_001, total number of reads: 53691864, uniquely mapped reads: 36570126, read length: 20-51, single-end
12-p10B-INP-22_S11_R1_001, total number of reads: 53108213, uniquely mapped reads: 36309933, read length: 20-51, single-end
13-bla-AZ460_S13_L001_R1_001, total number of reads: 42521054, uniquely mapped reads: 31353061, read length: 20-51, single-end
13-p7-INP-25_S12_R1_001, total number of reads: 65255674, uniquely mapped reads: 44992111, read length: 20-51, single-end
14-p12B-INP-27_S15_R1_001, total number of reads: 35234708, uniquely mapped reads: 23108870, read length: 20-51, single-end
15-INP-p12_S15_L001_R1_001, total number of reads: 37058754, uniquely mapped reads: 25866446, read length: 20-51, single-end
16-INP-NSN_S16_L001_R1_001, total number of reads: 35891024, uniquely mapped reads: 25632081, read length: 20-51, single-end
18-INP-SN_S17_L001_R1_001, total number of reads: 38589975, uniquely mapped reads: 26670228, read length: 20-51, single-end
19-INP-MII_S18_L001_R1_001, total number of reads: 38427374, uniquely mapped reads: 26191638, read length: 20-51, single-end
20-INP-8c_S19_L001_R1_001, total number of reads: 49189660, uniquely mapped reads: 35710834, read length: 20-51, single-end
21-INP-mor_S20_L001_R1_001, total number of reads: 33198212, uniquely mapped reads: 23702087, read length: 20-51, single-end

22-INP-bla_S21_L001_R1_001, total number of reads: 49776222, uniquely mapped reads: 34934961, read length: 20-51, single-end
1-100kAZstd_S1, total number of reads: 25888988, uniquely mapped reads: 20613350, read length: 20-76, single-end
2-10kAZstd_S2, total number of reads: 25206305, uniquely mapped reads: 20024649, read length: 20-76, single-end
5-5-500-AZ-oc_S5, total number of reads: 27348682, uniquely mapped reads: 18718384, read length: 20-76, single-end
5-AZINP_S5, total number of reads: 39440815, uniquely mapped reads: 28894172, read length: 20-76, single-end
7-7-1k-AZ-oc_S7, total number of reads: 28366803, uniquely mapped reads: 20004662, read length: 20-76, single-end
1-MC-ChIP1-2AZ-p10-80-20250804_S1_L001_R1_001, total number of reads: 69128234, uniquely mapped reads: 19326332, read length: 151, single-end
2-MC-ChIP2-2AZ-p10-80-20250804_S2_L001_R1_001, total number of reads: 62967580, uniquely mapped reads: 19272187, read length: 151, single-end
3-MC-ChIP1-2AZ-Zyg-200-20250804_S3_L001_R1_001, total number of reads: 67470354, uniquely mapped reads: 17144099, read length: 151, single-end
4-MC-ChIP1-2AZ-2c-200-20250804_S4_L001_R1_001, total number of reads: 49797213, uniquely mapped reads: 14197582, read length: 151, single-end
5-MC-ChIP1-2AZ-4c-200-20250804_S5_L001_R1_001, total number of reads: 72470298, uniquely mapped reads: 21438235, read length: 151, single-end
6-MC-Input1-2AZ-p10-80-20250804_S6_L001_R1_001, total number of reads: 221005827, uniquely mapped reads: 71474036, read length: 151, single-end
7-MC-Input2-2AZ-p10-80-20250804_S7_L001_R1_001, total number of reads: 363926419, uniquely mapped reads: 153751135, read length: 151, single-end
8-MC-Input1-2AZ-Zyg-200-20250804_S8_L001_R1_001, total number of reads: 291658776, uniquely mapped reads: 219112483, read length: 151, single-end
9-MC-Input1-2AZ-2c-200-20250804_S9_L001_R1_001, total number of reads: 261521114, uniquely mapped reads: 191031415, read length: 151, single-end
10-MC-Input1-2AZ-4c-200-20250804_S10_L001_R1_001, total number of reads: 167020614, uniquely mapped reads: 153193486, read length: 151, single-end

Antibodies

H2A.Z cat# 39114, Active Motif Lot. 06217001

Peak calling parameters

Default settings in EaSeq (windowsize: 100bp, p-value: 1E-5, FDR: 1E-5, Log2fd: 2, Merge within: 100 bp, Fragment sizes: 150 bp) using paired input samples as negative controls.

Data quality

Data quality before and after trimming was assessed by FastQC v0.12.1 (https://www.bioinformatics.babraham.ac.uk/projects/fastqc/). To match the read length single-end fastq files were trimmed to 51bp using Trim Galore v.0.6.7 (https://github.com/FelixKrueger/TrimGalore) with the parameter –hardtrim5 51. To remove adapters and low basecalling sequence, single-end fastq files were trimmed using Trim Galore v0.6.7 with the parameter -novaseq 20. Trimmed reads were then mapped to mm10 using bowtie v1.3.0 (Langmead et al., 2009) with the parameter -m 1 to remove multimapping reads from the reported output.
For peak calling a very strict FDR threshold of 1E-5 was used (0.00001), and all peaks with less than 4 fold difference in normalized enrichment were not reported. This threshold resulted in more than 99% of the peaks having a 5-fold enrichment or better.

Software

ChIP-Seq data was analyzed using an in-house Nextflow pipeline (https://github.com/lerdruplab/ew-qctrimalign) written according to nf-core guidelines. Briefly, the pipeline was executed using Nextflow v23.4.1. Data quality before and after trimming was assessed by FastQC v0.12.1 (https://www.bioinformatics.babraham.ac.uk/projects/fastqc/). Single-end fastq files from P10 (C57BL6/N), zygote, 2-cell and 4-cell samples were hard-trimmed to 51bp to match the read length of the remaining samples using Trim Galore v0.6.7 (https://github.com/FelixKrueger/TrimGalore) with the --hardtrim5 51 parameter. Quality trimming was performed using Trim Galore v0.6.7 with the parameter -novaseq 20. Trimmed reads were then mapped to mm10 using bowtie v1.3.0 with the parameter -m 1. Obtained bam files were sorted using samtools v1.2 and converted to bed format using bedtools v2.31.1. Bed files were then used for downstream analysis in EaSeq.
